# System Dynamics Analysis of Construction Safety Risk considering Existing Railway Lines

**DOI:** 10.1155/2022/1256975

**Published:** 2022-02-18

**Authors:** Xiaoye Zeng, Naixin Huang, Yang Han, Yang Yin, Jianling Huang

**Affiliations:** ^1^Department of Engineering Management, School of Civil Engineering, Central South University, Changsha 410083, Hunan, China; ^2^College of Design, Construction and Planning, University of Florida, Gainesville, 32601, FL, USA

## Abstract

Existing railway line (ERL) construction safety has received significant attention during the past decades due to the high accident rate and the difficulty of progress development under the limited synthesis construction time schedule (SCTS). However, the previous literature is dominated by the construction safety of new railway lines, while research on construction safety of ERLs is limited. This paper analyzed the interactions and causal relationships between construction safety risk (CSR) and multiple factors and classified feedback loops. Hence, a system dynamics model was developed, and a series of tests were conducted to simulate the evolution of CSR under different group environments. The results indicated that (1) the CSR considering ERLs is significantly relevant to the implementation degree of SCTS. For situations where there are more delays and more schedule pressure, construction safety accidents tend to have a higher level. (2) Work efficiency is negatively related to construction safety accidents probability. The increase of work intensity could reduce schedule pressure in the short term but could increase construction safety risk in a long time. Applying both appropriate work efficiency and work intensity may achieve an acceptable result. This paper adds to the knowledge of construction safety risk management in terms of implementation and offers lessons and references for future construction safety management considering ERLs.

## 1. Introduction

With the great improvement in train speed and operating conditions, some new railway projects will inevitably be built close to the existing railway lines (ERLs) [1]. Under the background of uninterrupted operation, to ensure the transportation of ERLs, the construction of new railway projects cannot be arranged randomly. It can only operate within a certain time called synthesis construction time schedule (SCTS) [2]. The SCTS is the time reserved in the train operation diagram, specifically used for the construction. No trains will be running during that time. The duration of SCTS should be more than 180 minutes when the construction is near a normal railway and 240 minutes at 0:00–6:00 when the construction is nearby a high-speed railway [3]. When the SCTS is long, the fluency of the transportation organization will be affected, thus affecting the efficiency of the operation of ERLs. On the contrary, if the SCTS is too short, the normal construction schedule will be restricted, affecting the efficiency of construction operation and increasing the construction cost [4]. Due to the mutual interference between the construction of new railway projects and transportation of ERLs, the effective construction time and space are more and more limited, and the construction safety issues are more prominent [5]. Thus, it is challenging and demanding to ensure construction safety under the background that the transportation organization of ERLs affects the construction organization of the new railway projects.

Each country has different management modes for the construction and maintenance of ERLs. In Japan, for the traffic density of Shinkansen during the daytime, except for necessary patrol inspection, all construction and maintenance work will be arranged within the SCTS of 12:00∼6:00 am, and all trains in the SCTS will be suspended. 12:00∼3:00 am is the time for construction or maintenance, and 3:00∼6:00 am is the time of inspection and acceptance [6]. Construction and maintenance also adopt the 6 hours of SCTS in France, it is generally during 11:30 pm∼5:30 am, and the actual operation time is controlled under 4 hours [7]. High-speed railways and general-speed railways are interlinked alternately into networks in Germany, and the railway lines are mixed passengers and freight trains. For the density and trains that run at night, the SCTS is arranged at 3:30∼6:00 am. Only one line is blocked, and the other line is running normally. The express freight trains pass before the SCTS hours, during which only a small number of slow freight trains run. More construction and maintenance work will be carried out on weekends with no train traffic [8]. SCTS of construction and maintenance of ERLs in the above countries takes a long time, and there are no trains or only a few single lines of freight trains running during the SCTS. Transportation has a negligible impact on construction safety, and the construction safety of ERLs is not prominent as that of China. The setting of SCTS has constraints and impacts on the operation and the passing capacity of the railway. If the time of SCTS is too short, it cannot guarantee the completion of the construction task and will affect the quality and efficiency of the construction work, thus affecting the construction schedule.

Safety accidents during construction have been and continue to be a global problem [9], and they can cause casualties and huge economic losses [10]. Generally speaking, accidents are raised by many interacting systemic factors [11]. An accident or an unsafe event may be caused by some elusive causes [12, 13]. Since Heinrich realized that unsafe behavior was the dominant cause of safety accidents [14], there has been a growing amount of research concerning their contribution to accidents in recent years. Bird revised Heinrich's theory and proposed that the essential cause of accidents depends on management. Accidents were more likely to be caused by “unsafe acts of people” and “unsafe mechanical or physical conditions” [15, 16]. The “unsafe acts of people” and “unsafe physical or mechanical conditions” are collectively referred to as on-site errors. Management errors are the deep-seated reason that causes safety accidents [17]. Unsafe behavior can be defined as intentional or unintentional violations of safety compliance expectations [18]. An unsafe condition is when the physical layout, tools, equipment, and/or materials in a workplace or work location violate contemporary safety standards [19]. Avoiding unsafe behaviors and unsafe conditions can be effective in reducing the probability of safety accidents [20]. The majority of existing studies focus on the safety accidents in independent new rail projects [21, 22], and few studies consider CSR in the presence of ERLs. Furthermore, given the huge mediating effect of CSR considering ERLs, the mechanism of how CSR changes under the influence of multiple factors needs more attention. However, the evolution mechanisms of CSR considering ERLs are not yet clear, and it is necessary to be explored further. To fill the aforementioned research gap, this paper aims to investigate the laws and effects of influencing factors in the safety management system considering ERLs, establishing a scientific causal model, and simulating the evolution of the system under different conditions to provide guidance for management decisions.

System dynamics is a system simulation method for analyzing production management and inventory management created by Jay Forrester and originally named Industrial Dynamics [23]. It has often been used for analyzing and understanding complex safety problems [24]. For example, researchers adopt SD to gain insight into the cause of major accidents [25–27]. SD was applied to gain insight into the complexity and coupling of project elements [28] and was shown as an efficient tool to simulate the dynamics of safety attitudes and behaviors [29] and to organizational learning [27, 30, 31]. As a systematic approach, SD emphasizes the feedback between variables in a system and understanding the behavior and dynamics of complex systems with time [32]. Therefore, it was used as a theory development tool [33]. SD emphasizes the holistic nature of systems and the nonlinear characteristics of complex systems and considers that the behavior patterns and characteristics of a system depend mainly on the internal dynamic structure and feedback mechanisms. The system develops and evolves according to certain laws under the action of internal and external dynamics and constraints. The approach of system dynamics to complex problems is a combination of qualitative and quantitative, and holistic and analytical thinking. It is a method of analysis, synthesis, and reasoning. This paper uses SD to investigate the complexity and coupling of project elements (e.g., construction schedule, safety cost, and safety) from a system thinking perspective considering ERLs.

The paper is organized as follows.  Section 2 analyzes the relevant factors that influence the construction safety of new projects near ERLs and sets up the causal model.  Section 3 sets up the SD model of construction safety considering ERLs, uses data collected from a construction site, and validates the model. In Section 4, tentative data are used to simulate the relationship between these variables and the occurrence of accidents. The results are analyzed and discussed to understand the dynamics of the management components. Finally, Section 5 provides concluding remarks.

## 2. Relevant Factors Influence on Construction Safety of ERLs

### 2.1. Identification of Feedback Loop for Construction Safety

This study is based on unsafe behaviors and unsafe conditions in those two perspectives and only considers the construction progress, cost, and safety factors. Other relevant factors were not considered. This study identifies and refines the factors affecting construction safety and analyzes the relationship between the various factors to establish a causal model. It can visually show the influence of each factor on construction safety and make the relationship among each factor clear. This paper made use of a large amount of literature involving railway construction safety in recent years to summarize while forming a preliminary understanding of the identification of railway construction safety impact factors considering ERLs. Literature was based on Google Scholar, Elsevier, Web of Science, and Scopus, searched by entering keywords such as railway construction safety. Descriptions of construction safety influence factors considering ERLs in the literature were carefully extracted. According to the literature, the driving factors of construction safety can be categorized as follows:

1. Construction schedule: if the schedule of the railway construction was out of control, economic and social losses would be inevitable [34]. It is important to note that excellent schedule management does not mean compressed schedules, which can lead to safety incidents [35, 36]. Management measures that are taken to expedite production put workers under pressure to increase productivity, which is negatively impacted[37]. When production pressure (e.g., excessive workload, required work pace, and time pressure) is perceived, workers perceive increased risks and barriers, leading them to be more likely to work with unsafe behaviors [38]. On the contrary, construction safety accidents are likely to cause project delays [39]. Therefore, previous studies have shown that schedule pressure is the critical link between scheduling and safety in construction.
2. Cost: this paper does not consider the total production cost during the construction. It only analyzes the safety investment within the boundary range of the model. Safety investments generally refer to funds spent on workplace injury prevention measures or activities that aim to protect workers' health and physical integrity [40, 41]. In many previous studies, the components of safety investment have been discussed, such as safety training, safety supervision, and safety protection [42–44]. Since the vast majority of those engaged in the grassroots work of railway construction are migrant workers, they are poorly educated and lack safety awareness [45]. Safety training improves the safety awareness of workers to a certain extent. Safety supervision is a reflection of the management's attitude towards unsafe behaviors [46]. The more strict the supervision of safety managers at the construction worksite, the more the probability of discovering unsafe behaviors [47]. Severe punishment for unsafe behavior can also serve as an effective warning [48]. Not using personal protective is one of the leading phenomena of unsafe behaviors [49]. In view of the high incidence of falling accidents in railroad construction, safety measures can be effectively taken to reduce the rate and severity of accidents [50]. Providing safety protection is a crucial complement to other safety measures (e.g., safety training) and is considered a last resort for hazard control measures for workers [51].
3. Safety subjects: the research on accident causation theory was pioneered by Heinrich [14], who analyzed 75,000 accident reports and developed a domino theory (model) of accident causation. The analysis led him to conclude that 88% of accidents are caused by unsafe behavior and 10% of accidents are caused by unsafe conditions. Unsafe behaviors and unsafe conditions can directly lead to the occurrence of accidents [52]. According to past statistics in China, more than 80% of accidents were caused by workers' unsafe behaviors [53]. Unsafe conditions on a construction site are events that are not related to people and are a natural part of the initial construction site conditions [19].

### 2.2. Causality Analysis of Construction Safety

**Figure 1:**
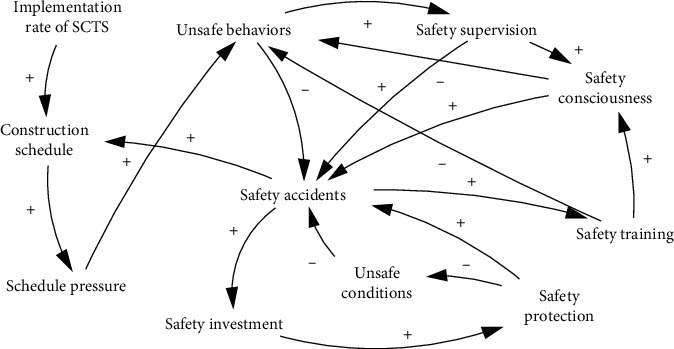
SD modeling for construction safety considering ERLs.

In order to understand the causal relationships of construction safety considering ERLs, the causal loop diagram is proposed, as shown in Figure 1 (the polarities represent a positive or negative impact between variables). Due to the SCTS being fixed and limited, the construction must be executed strictly according to the plan. Owing to the complexity and uncertainty of the construction environment and conditions, schedule delays are inevitable [54–56]. The resulting schedule pressure (e.g., being pressed to work faster) leads to unsafe behaviors [57, 58]. Accidents caused by unsafe behaviors, in turn, lead to delays in production, thus creating production pressure [59]. With increased investment in safety comes increased safety training, supervision, and protection. Safety training and safety supervision are positively correlated with the improvement of safety consciousness and behaviors [60, 61]. Insufficient safety protection leads to the deterioration of cumulative unsafe conditions in the worksite and eventually to accident occurrences, which increases safety investment [62, 63]. In summary, the model reveals these feedback processes, and it is vital to consider schedule, safety cost, and safety on an operational level.

## 3. Modeling

System dynamics modeling is a typical simulation method, focusing on the interaction between factors of complex systems [64]. It predicts the changing trend of the system and summarizes the dynamic development law of the system by constructing a causal feedback loop to describe the dynamic adjustment process of construction safety considering ERLs, which is an ideal research method. System dynamics modeling requires three steps: determining the system boundaries, constructing the model structure, and quantifying the action paths. The analysis of feedback loops and simulation experiments are typical research methods.

### 3.1. System Boundary

The SCTS is the most important element in the construction of ERLs; the cost that construction enterprises pay special attention to is taken as the reference elements, while other elements such as quality and environment are not taken as the analysis object. According to the causal model of construction safety, safety accidents (SA), safety training (ST), safety investment (SI), safety supervision (SS), safety protection (SP), SCTS, schedule pressure, fatigue, unsafe conditions (UC), unsafe behaviors (UB), construction schedule (CS), and safety consciousness (SC) are defined as main systematic variables. This study focuses only on the relationships and interactions among the eleven variables.

### 3.2. Model Structure

**Figure 2:**
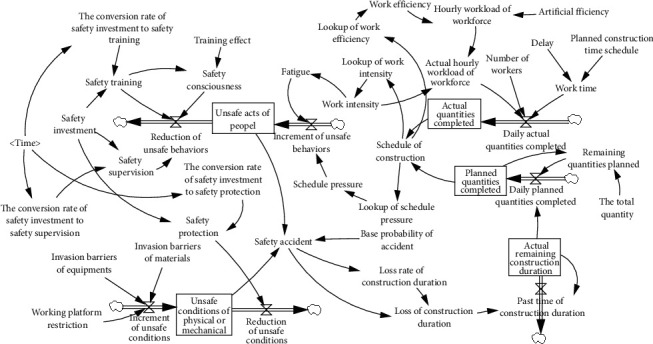
dynamics model of construction safety considering ERLs.

Based on the causal model of construction safety under the context of ERLs, we further defined the stock, flow, and feedback loop in the dynamic system. A stock is the value or level of the core variables accumulated in a dynamic system that reflects the system's changing state. According to the dynamic regulation system of construction safety considering ERLs, the system stocks are the UB level of people and the UC level of physical or mechanical. A flow is an activity that changes the stock. In this study, the flows are the decrease and increase of UB of people as well as the decrease and increase of UC of physical or mechanical. According to the causal model, the reduction and improvement of UB are related to dynamic variables such as ST, SC, SS, fatigue, and schedule pressure. The improvement and reduction of UC are associated with dynamic variables such as SP, working platform restriction, and invasion barriers of equipment and materials. In addition, the auxiliary variables, such as working platform restriction and invasion barriers of equipment and materials which reflect the UC, are closely related to the dynamic regulation of SA and need to be defined separately. In conclusion, the structure of the dynamics model of construction safety considering ERLs is shown in Figure 2.

### 3.3. Model Hypothesis and Path Quantification

Construction safety considering ERLs may be interfered with by many factors. It is hard to enumerate and quantify all the interactions between variables. The rational assumptions of the system dynamics model allow itself to focus on the vital variables and their influence paths while ignoring the interference of other unimportant and small probability events, thereby significantly reducing the complexity of the algorithm. In order to standardize the study, five hypotheses are proposed: (1) Only consider the relationship between unsafe behaviors, unsafe conditions, and safety accidents in the construction stage under the background of ERLs. (2) It does not consider the total production cost during the construction and only analyzes the safety investment. (3) The variables, such as total quantity, remaining quantities planned, daily actual quantities completed, daily planned quantities completed, actual quantities completed, planned quantities completed, and construction schedule, should be uniformly treated according to the progress unit. For example, the total quantity is 12,000 m, and the constructions schedule is 50 m/d. (4) Delay is set as a random function, and it is assumed that the delay occurs randomly. (5) Different lines have different actual situations. The values of relevant variables in this paper are derived from the production data of the case. The assigned values of correlation coefficients are the average values obtained after statistical analysis after collecting actual data.

Based on the above model assumptions, some mathematical functions are needed to describe the relationship between the key variables. Planned quantities completed (PQC) is the accumulation of daily planned quantities completed (DPQC); DPQC is related to planned quantities (PQ) and construction period (CP), which is an IF THEN ELSE function, and the specific description is shown in Table 1, so PQC is calculated as follows:

(1) $$\begin{array}{c} PQC=\overset{CP}{\underset{1}{\sum }}\text{DPQC}. \end{array}$$

Similarly, the mathematical function of the calculation related to the actual quantities completed (AQC) can be obtained as formula (2), where daily actual quantities completed (DAQC) is related to the following three variables: WN stands for the number of workers per day, AHWW stands for the actual workload of workforce per hour, and WT stands for the work time per day, so the mathematical expression of DAQC is shown in formula (3).

(2) $$\begin{array}{c} AQC=\overset{CP}{\underset{1}{\sum }}DA\;QC. \end{array}$$

(3) $$\begin{array}{c} \text{DAQC}=WN\times AHWW\times WT. \end{array}$$

In the above formula, WT is actually determined by planned SCTS and delay per day. The detailed description is shown in Table 1, where the delay is a random function whose output is 0 or the actual delay time. Different delays are set to explore the influence of transportation organization of ERLs on construction safety in this study.

The increment of unsafe behaviors (UBI) is related to the following two variables: fatigue stands for the count of those unsafe behaviors caused by workers' fatigue, SP stands for the count of those unsafe behaviors caused by the schedule pressure of workers. Then, the decrement of unsafe behaviors (UBD) is related to ST, SC, and SS, which stand for the conversion of the safety investment to safety training, safety consciousness, and safety supervision, respectively. So the mathematical expression of the level of unsafe behaviors (UBL) is shown in formula (4), and the mathematical expression of the UBI and UBD is shown in formulas (5) and (6), where *α*_1−5_ is the influence weight of each variable.

(4) $$\begin{array}{c} \text{UBL}=\text{UBI}-\text{UBD}. \end{array}$$

(5) $$\begin{array}{c} \text{UBI}=\alpha_{1}F+\alpha_{2}SP. \end{array}$$

(6) $$\begin{array}{c} \text{UBD}=\alpha_{3}ST+\alpha_{4}SC+\alpha_{5}SS. \end{array}$$

Correspondingly, the increment of unsafe conditions (UCI) is related to the following three variables: MBI stands for the materials beyond the boundaries set for transportation safety, EBI stands for the equipment beyond the boundaries set for transportation safety, and WPR stands for the restrictions on work platforms set to ensure transportation safety. In this study, the decrement of unsafe conditions (UCD) is related to the SP, which refers to the conversion of the safety investment to safety protection. Therefore, the mathematical expression of the level of unsafe conditions (UCL) is shown in formula (7), and the mathematical expression of the UBI and UBD is shown in formulas (8) and (9), where *β*_1−3_ is the influence weight of each variable.

(7) $$\begin{array}{c} UCL=UCI-UC. \end{array}$$

(8) $$\begin{array}{c} \text{UCI}=\beta_{1}\text{MBI}+\beta_{2}\text{EBI}+\beta_{3}\text{WPR}. \end{array}$$

(9) $$\begin{array}{c} \text{UCD}=SP. \end{array}$$

The unsafe behaviors and unsafe conditions actually determine safety accidents (SA), but the former two are necessary not sufficient conditions for the latter. *γ*_*s*_ is set as a constant standing for the base probability of an accident. Therefore, SA is calculated as follows:

(10) $$\begin{array}{c} SA=\gamma_{S}\left(\text{UCL}+\text{UBL}\right). \end{array}$$

All the previous formulas are the main mathematical functions in this study; more descriptions of variables and constants are shown in Table 1.

### 3.4. Model Validation

The validity tests of the model include mechanical error tests, dimensional consistency tests, and extreme condition tests. The model was built with Vensim in this study, which has passed the mechanical error tests, dimensional consistency tests, and extreme condition tests. The system model in this study included 35 variables and 10 constants. The main variables and function relationships in the model are described in Table 1.

**Figure 3:**
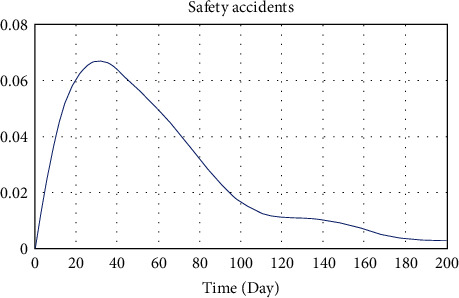
Simulation result of safety accidents probability of the case.

Workers in the real world are faced with different situations every day and therefore make decisions accordingly. The conditions of the workplace vary day to day. As time goes on, the decision of workers and conditions of the workplace may be changed. To simulate this, the model sets some events (safety training, safety supervision, working platform restriction, etc.), which are triggered every day. According to the functions we set above, unsafe behaviors were performed, and unsafe conditions were accumulated. Consequently, the time step of the model was set to 200 days to ensure that a long enough observation time was available to show all possible trends. The initial value of safety accidents probability was set as 0. Vensim 8.2 was used to simulate the system dynamics in this study. And the input parameters of a railway cutting project close to the ERLs were designed. The main input parameters are shown in Table 3 and were determined by interviewing our industry workers involved in the data collection or based on available research literature and practical survey data. Relevant parameters were input into the model to conduct simulation analysis on the relationship between SCTS, construction schedule, UB, UC, and SA of the project. The simulation results are shown in Figure 3.

The construction risk degree and grade standards of existing lines were set by referring to “Technical Regulations for Risk Management of Railway Construction Engineering and Relevant Regulations,” as shown in Table 2. The goal of the system safety management level was set as low risk.

Figure 3 shows that the safety accidents probability of the project reaches the maximum on the 31st day, which was a high-risk degree according to Table 2. It was reduced to medium risk on the 82nd day and low risk on the 187th day. Combined with the actual situation of the case project, it was found that the simulation result of the system dynamics was basically the same as the actual situation. That is, with the increase of construction schedule, unsafe behaviors and unsafe conditions rose to a high level at the end of the first month, which led to the rise of safety accidents. However, the safety investment came into play simultaneously. Various safety measures reduced the unsafe behaviors and unsafe conditions, which led to the reduction of safety accidents, so the safety risk was reduced to a low level on the 187th day. Figure 3 also indicates that the risk level would not decrease indefinitely with the increase of safety investment, which means there is no absolute zero safety risk. In general, it can be seen that the internal relationship among the parameters, the equations, and the parameters established in the system dynamics model established in this study are reasonable and can reflect the actual situation of engineering management.

## 4. Simulation and Discussion

### 4.1. Simulation Analysis in Different Delay

**Figure 4:**
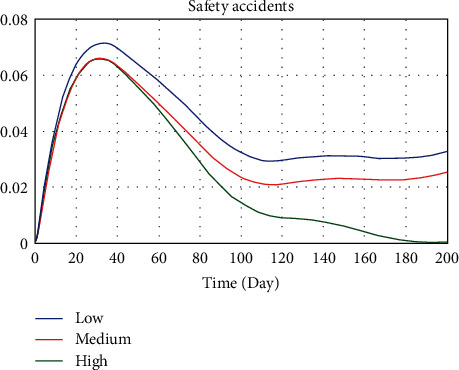
Comparison of safety accidents probability under different implementation degrees of SCTS.

**Figure 5:**
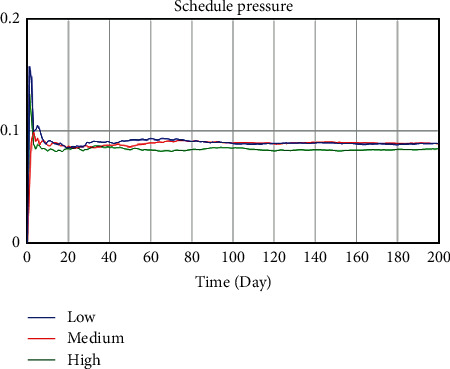
Comparison of schedule pressure under different implementation degrees of SCTS.

**Figure 6:**
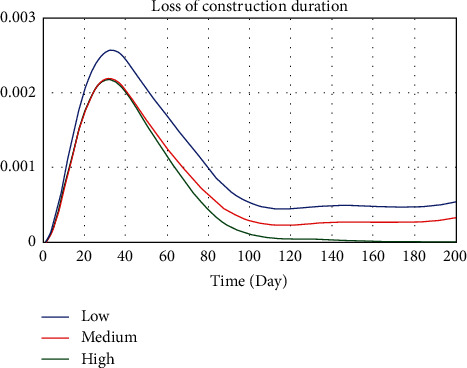
Comparison of safety accidents probability under different implementation degrees of SCTS.

The influence of SCTS on safety accidents is essentially reflected in the work time. Although the time of SCTS is planned, which means the work time is fixed, delay on ERLs is inevitable, resulting in frequent changes of work time. To observe the correlation between SCTS and safety incidents, different assignments of delay are shown in Table 4. Different assignments of delay correspond to different implementation degrees of SCTS. The smaller the assignment, the higher the implementation degree. The simulation experiment results are shown in Figures 4−6.

From Figure 4, it can be observed that when the implementation degree of SCTS is high, the safety accidents probability of the project reaches the maximum on the 32nd day, and the valve is 0.066. It is reduced to medium risk on the 79th day and low risk on the 165th day. Eventually, the safety accidents probability is infinitely close to 0. Correspondingly, when the implementation degrees of SCTS are medium and low, the safety accidents probability of the project reaches the maximum on the 32nd and 33rd days, and the valves are 0.0663 and 0.0772. It is reduced to medium risk on the 87th day and 108th day and kept at the medium risk level until the 200th day. From Figures 5 and 6, it can be seen that, with the increase of implementation degree, schedule pressure and loss of construction period are raised obviously. The reason behind this is that when the implementation degree of SCTS is low, the available working hours are limited. Due to the fact that the daily workload is planned, the schedule pressure increases rapidly. However, the workers will adjust the construction schedule according to the working hours and daily workload. As time goes on, the schedule pressure decreases and begins to level off. Loss of construction period is related to the safety accidents probability. A safety accident could lead to a construction shutdown and then result in loss of construction period.

### 4.2. Simulation Analysis in Different Safety Investment

**Figure 7:**
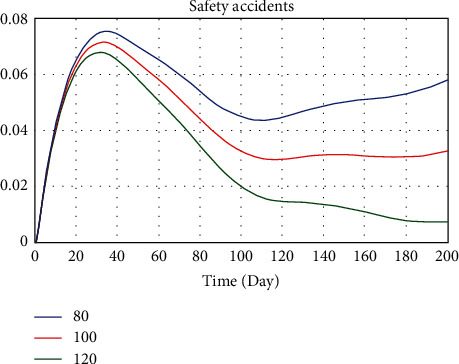
Comparison of safety accidents probability under different safety investments.

Using the three major components categorized by existing researches [40, 65–67] as a point of departure, we chose to study three safety investments: (1) implementation of innovative technological tool proactive protection system, (2) employment of safety supervisor for conducting inspections, and (3) encouragement on being responsible for the safety of themselves and other coworkers with safety training. Three different safety investment assignments of 80, 100, and 120 (unit: CNY/person·d) were set to observe the relevance between safety investment and safety accidents. The simulation experiment result is shown in Figure 7.

The result from the simulation demonstrates the effectiveness of safety investment on construction safety risk. From Figure 7, it can be observed that when the safety investment is 120 CNY/person·d, the safety accidents probability of the project reaches the maximum on the 32nd day, and the valve is 0.0679. Correspondingly, when the implementation degree of SCTS is medium and low, the safety accidents probability of the project reaches the maximum on the 33rd and 35th day, and the valves are 0.0716 and 0.0756. In brief, higher safety investment may result in better safety performance.

### 4.3. Simulation Analysis of Construction Strategy

**Figure 8:**
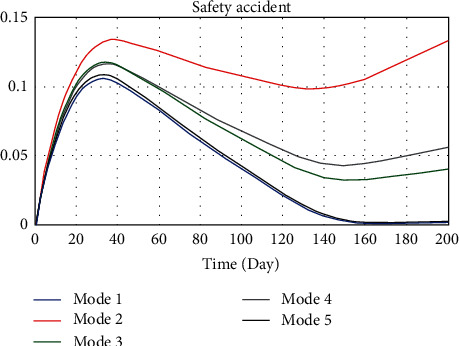
Comparison of safety accidents probability under different implementation degrees of SCTS.

**Figure 9:**
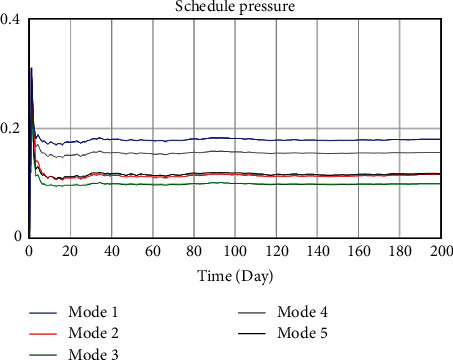
Comparison of safety accidents probability under different implementation degrees of SCTS.

**Figure 10:**
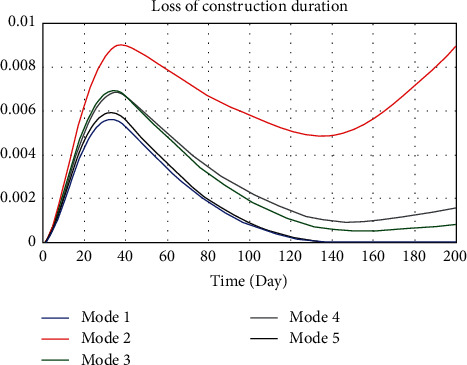
Comparison of safety accidents probability under different implementation degrees of SCTS.

Improving work efficiency and increasing work intensity are common means to reduce schedule pressure. To observe the influence of those two factors on the whole model, 5 modes were set, respectively, as shown in Table 5. Different value assignments represented different strategies. Work efficiency and intensity in mode 1 were selected as 1.5 and 0, indicating that the construction was efficient and the work was easy. It would not cause physical and psychological discomfort to the workers. Mode 1 was the ideal mode. Similarly, mode 2 was the crushing mode, modes 3 and 4, respectively, represent some preference mode, and mode 5 was the equilibrium mode. That is, different modes represent different strategies. When other variables remain the same, the dynamics model simulates the evolution by changing the value assignments of the two variables mentioned above. According to the safety accidents probability, change of schedule pressure and loss of construction period after system runs to make rational decisions. The simulation results are shown in Figures 8–10.

According to the comparative analysis of Figures 8–10, mode 1 has the lowest safety accidents probability and loss of construction period but the highest schedule pressure. On the contrary, mode 2 has the most downward schedule pressure and the most increased safety accidents probability and loss of construction period. The result shows that it is unscientific to unilaterally improve work intensity to speed up the progress under the schedule pressure. This practice should be avoided in the construction of ERLs. Advanced construction equipment and scientific construction methods should be actively promoted to improve construction efficiency. The construction of ERLs should be carried out in a planned and organized way. However, mode 1 can only be used as an ideal mode in management practice. Improving construction efficiency is limited under the current construction technology and method, and other satisfactory solutions can only be sought.

Compared with other modes, the safety accidents probability, schedule pressure, and loss of construction period of modes 3 and 5 all maintain low values, which can be a satisfactory solution. The safety accidents probability of mode 5 is relatively lower, and mode 3 performs better in terms of schedule pressure. However, the progress pressure of mode 4 in the first week is slightly lower than that of mode 3 and mode 5 and then increases significantly; the safety accidents probability and loss of construction period are also at a high level. It indicates that the strategy based on increasing work intensity can only accelerate the progress in the short term. When the fatigue degree of personnel reaches a certain value, the safety accidents probability will be increased, which will interfere with the progress and cause economic losses of accidents and rework.

## 5. Discussion

The results from the simulations demonstrate that the schedule pressure after stabilization and loss of construction period are proportional to the implementation degree of SCTS. With the decrease of implementation degree of SCTS, the schedule pressure, loss of construction period, and safety accidents probability all increase to different degrees; especially, the safety accidents probability changes significantly. It can be seen that the evolution of the implementation degree of SCTS has a significant influence on the safety accident probability. To effectively enhance and improve the construction safety management of ERLs, the transportation organization should be optimized, and the implementation degree of SCTS should be improved.

Different safety investments have different effects on the safety accident probability, and safety investments have a positive effect on accident prevention. Good safety training not only raises the level of risk awareness but also persuades individuals to be less tolerant of risks. The findings of this study further showed that the interventions that combine good safety supervision with safety protection are more likely to reduce safety accident probability.

When the schedule pressure is low, it can be preferred to ensure safety. Increasing a certain amount of work efficiency can accelerate the schedule by reasonably arranging the working platform and optimizing equipment and processes. When the schedule pressure is high, increasing work intensity and work efficiency simultaneously is better. Under the condition of ensuring the low safety accidents probability, parallel construction can be organized as far as possible to expand the working platform. At the same time, construction organization and safety protection should be optimized to avoid interference between working platforms and construction procedures. When the construction period is nearing the end, appropriate consideration can be made to increase the work intensity, which can avoid the safety accidents and loss caused by fatigue accumulation in the later period and complete the planned project faster.

## 6. Conclusions

Based on the causal model of construction safety considering ERLs, a system dynamics model is proposed to simulate the construction safety evolution process, and a series of simulations are performed under different conditions. The results confirmed that the construction safety considering ERLs is significantly relevant to the implementation degree of SCTS. The incidence of construction safety incidents tends to be higher because the greater the possibility of construction delays, the greater the schedule pressure. However, even in each level of construction safety accidents probability, the evolution curve reached the maximum at about one month. As the countermeasure took effect, the evolution curve declined after reaching the maximum value and finally stabilized. Work efficiency has a negative impact on construction safety accidents probability. The increase of work intensity could reduce schedule pressure in the short term. Still, due to psychological and physiological factors, unsafe behaviors increased in a certain range. Interestingly, there is a marginal cost effect on the effect of work intensity on schedule pressure. The improvement in schedule pressure is not significant when work intensity increases to a certain level.

The main contribution of this study is that it is a major step forward in integrating system dynamics and safety in the examination of construction safety management considering the ERLs. Theoretically, rather than studying construction safety considering ERLs in a static manner, this study examined the evolution as it unfolds over time. This study treats construction safety management considering the ERLs as a dynamic problem caused by “system structure” and a complex phenomenon. In this system, unsafety behaviors and unsafety conditions have significant impacts on construction safety risk considering ERLs. In contrast, almost all existing studies focus on construction safety risks relevant to new railway projects, with little research on construction safety management considering ERLs. Methodologically, a system dynamics approach can provide a rich dynamic perspective to visually explain the causes of construction safety risk changes. In general, this paper complements the body of construction safety management considering the ERLs in terms of theoretical foundations and implementation and offers references and lessons for the design and operation of construction safety management in the railway construction industry in the future.

The limitation of this study is the simplification of the interactions involved in construction safety. In fact, the mutual impacts are very complex, far more than those proposed in the hypothesis of this study. In fact, the vast majority of construction safety accidents are caused by people's unsafe behaviors. Furthermore, workers' hazard perception does play an essential role in the construction safety management considering ERLs. In the future, “how does the worker's hazard perception impact construction safety considering ERLs dynamically” should be studied further.

## Figures and Tables

**Table 1:** Principal variables and constants description of the SD model.

Variables designation	Unit	Description
Schedule of construction	Dimensionless	IF THEN ELSE (actual quantities completed < planned quantities completed, (1− actual quantities completed)/ planned quantities completed, 0)
Schedule pressure	Dimensionless	1 ^*∗*^ WITH LOOKUP (construction schedule ([(0, 0)-(1, 1)], (0, 0), (0.1, 0), (0.2, 0.1), (0.5, 0.7), (0.7, 1), (1, 1))
Work intensity	Dimensionless	1 ^*∗*^ WITH LOOKUP (construction schedule ([(0, 0)-(1, 1)], (0, 0), (0.2, 0.3), (0.4, 0.5), (0.6, 0.7), (0.8, 0.9), (1, 1))
Fatigue	Dimensionless	WITH LOOKUP (work intensity ([(0, 0)-(1, 1)], (0, 0.045), (0.1, 0.21), (0.17, 0.3), (0.25, 0.37), (0.3, 0.4), (0.6, 0.4), (0.7, 0.45), (0.8, 0.58), (0.9, 0.76), (1, 1))
Reduction of unsafe behaviors	Dimensionless	0.33 ^*∗*^ safety training + 0.33 ^*∗*^ safety consciousness + 0.34 ^*∗*^ safety supervision
Increment of unsafe behaviors	Dimensionless	0.35 ^*∗*^ fatigue + 0.65 ^*∗*^ schedule pressure
Increment of unsafe conditions	Dimensionless	0.38 ^*∗*^ working platform restriction + 0.31 ^*∗*^ invasion barriers of equipment + 0.31 ^*∗*^ invasion barriers of material
Safety accidents	Dimensionless	Safety accidents probability (shown as Table 2), base probability of accidents ^*∗*^ (unsafe behaviors + unsafe conditions)
Loss of construction period	d	Safety accident ^*∗*^ loss rate of construction period
Daily actual quantities completed	m	Work time ^*∗*^ actual hourly workload of workforce ^*∗*^ number of workers
Daily planned quantities completed	m	IF THEN ELSE (actual remaining construction period ≤ 0, remaining quantities planned, remaining quantities planned/actual remaining construction period)
Hourly workload of workforce	m	Artificial efficiency × (1 + work efficiency)
Actual hourly workload of workforce	m	Hourly workload of workforce × (1 + labor intensity)
Work time	h	Planned construction time schedule−(delay/60)
Delay	min	RANDOM NORMAL (0, 20, 7, 15, 1)

**Table 2:** Degree and countermeasure for construction risk of existing railway lines.

Risk degree	Probability of casualty (0∼1)	Countermeasure
Low	<0.003	No measure
Medium	0.003∼0.03	Strengthen daily management and increase investment in safety
High	0.03∼0.3	Strengthen daily management and monitoring and increase investment in safety
Extreme high	>0.3	Measures must be taken to reduce risks and increase investment in rectification

**Table 3:** Main parameters of the model.

Parameter names	Parameter values	Parameter names	Parameter values
Total quantities	12,000 m	Planned construction time schedule	4 h
Number of works	100	Efficiency	0.075
Training effect	0.85	Safety investment	100 CNY/person·d
Working platform restriction	0.312	Invasion barriers of equipment	0.182
Invasion barriers of materials	0.236	Base probability of accident	0.03

**Table 4:** Assignment of different implementation rates of synthesis construction time schedule.

Implementation degree of SCTS	Assignment of delay
High	RANDOM NORMAL (0, 20, 8, 15, 1)
Medium	RANDOM NORMAL (0, 30, 10, 8, 1)
Low	RANDOM NORMAL (0, 40, 17, 10, 1)

**Table 5:** Value assignments under different strategies.

	Work efficiency	Work intensity
Mode 1	1.5	0
Mode 2	0	1.5
Mode 3	1.5	0.5
Mode 4	0.5	1.5
Mode 5	1	1

## Data Availability

All datasets generated for this study have been included in the paper.
